# Application of Molecular Dynamic Simulation in the Enantiorecognition Mechanism of the Pharmaceutically Relevant Leu‐Phe Dipeptides With Four Zwitterionic Chiral Stationary Phases

**DOI:** 10.1002/jssc.70220

**Published:** 2025-07-08

**Authors:** Ina Varfaj, Roccaldo Sardella, Yana A. Klimova, Leonid D. Asnin, Michael Kohout, Andrea Carotti

**Affiliations:** ^1^ Department of Pharmaceutical Sciences University of Perugia Perugia Italy; ^2^ Perm National Research Polytechnic University Perm Russia; ^3^ Department of Organic Chemistry University of Chemistry and Technology Prague Czech Republic

**Keywords:** enantiorecognition mechanism, HPLC, molecular dynamic simulations, zwitterionic *Cinchona* alkaloids‐based chiral stationary phases

## Abstract

In order to broaden the applicability of the molecular dynamics technique and to further validate the efficacy of a computational protocol recently developed in our laboratory, the present study aims to elucidate the enantiorecognition mechanisms involving four zwitterionic *Cinchona* alkaloid‐based CSPs under reversed‐phase (RP) conditions. In this study, we use the enantiomeric dipeptides D‐leucine‐D‐phenylalanine and L‐leucine‐L‐phenylalanine as probes to investigate the properties of CHIRALPAK ZWIX(+) and ZWIX(‐), as well as ZWIX(+A) and ZWIX (−A). The Leu‐Phe dipeptide has considerable potential in the pharmaceutical field due to its potential applications in drug delivery, therapeutics and as a building block for peptidomimetics. Furthermore, Leu‐Phe is one of the few uncapped dipeptides composed of natural amino acids capable of forming stable hydrogels.

The in silico protocol was successfully optimized by setting the simulation box size, run time, and number of frames to record to generate molecular dynamics trajectories as informative as possible. Importantly, the analyses were in complete agreement with the experimental EO, providing insights into the driving forces involved in the enantiorecognition mechanism. In particular, salt bridges and hydrogen bonds were confirmed as the primary interactions, while π–π and π–cation interactions were identified as complementary to facilitate the SO–SA association.

## 1 Introduction

The continuous advancement of computational hardware and software has facilitated the development of advanced molecular modeling techniques and methodologies, which are now being utilized in ways that were previously impractical. In particular, molecular dynamics (MD) simulations have been employed to elucidate the enantioselective mechanisms underlying the experimental elution order (EO). These computer‐aided investigations have been conducted using various types of chiral stationary phases (CSPs) and elution modes, yielding enhanced molecular‐level insights [1, 2, 3, 4, 5, 6, 7, 8, 9, 10]. While many of these methodologies have been successfully applied to HPLC data, the predictive capability of MD simulations is occasionally compromised by the oversimplification of the overall chromatographic system and the thermodynamic characteristics of the enantiorecognition process. Specifically, the entropic effects that are often critical to the enantioseparation process are frequently overlooked in these simulations.

In addition to MD simulations, docking strategies are also pertinent in this context. These protocols frequently provide valuable insights into the molecular mechanisms underlying enantiorecognition processes, thereby elucidating the interactions between the chiral selector (SO) and the enantiomeric select and (SA) complex.

A comprehensive understanding of enantiorecognition involving a specific CSP offers the additional advantage of identifying strategies for enhancing its enantioselectivity through targeted modifications of specific chemical attributes. Moreover, the ability to rapidly quantify the energetic differences of the enantiomeric associations with a CSP is instrumental in determining the optimal one for a given application. This consideration has significant implications for time efficiency and reductions in energy and reagent consumption from a green chemistry perspective, which also translates into economic benefits [11, 12].

In contrast to the docking technique, which typically identifies the most stable chiral selector–analyte (SO–SA) complex for each enantiomer, MD simulation allows for the consideration of a distribution of SO–SA complexes based on their energy profiles. This approach provides a more realistic representation of the events occurring within the chromatographic column. Furthermore, MD simulations encompass the entire chromatographic environment, including the silica layer, the underivatized silanols, and the silanols modified with chiral selector units. In addition, these methodologies explicitly account for the mobile phase, which is not merely a passive medium for the transport of analytes through the column but significantly influences the conformational preferences of both the enantiomeric SAs and the SO, thereby directly affecting the enantiorecognition process [1, 2, 3, 4, 5, 6, 7, 8, 9, 10].

**FIGURE 1:**
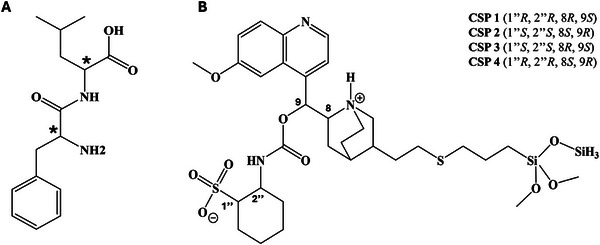
Structures of the compounds under investigation (A). Structure of the employed CSPs: CSP 1 and 3 incorporating a quinidine‐based chiral selector, CSP 2 and 4 incorporating a quinine‐based chiral selector (B). All CSPs are commercially available from Chiral Technologies Europe (Illkirch Cedex, France).

In order to broaden the applicability of MD techniques and further validate the efficacy of a computational protocol recently developed in our laboratory, the present study aims to elucidate the enantiorecognition mechanisms involving four zwitterionic *Cinchona* alkaloid‐based [10]. The investigated CSPs are CHIRALPAK ZWIX(+) and ZWIX(−), as well as ZWIX(+A) and ZWIX(−A) (Figure 1B), used under reversed‐phase (RP) conditions. In this study, we utilize enantiomeric dipeptides D‐leucine‐D‐phenylalanine and L‐leucine‐L‐phenylalanine (Figure 1A) as probes. The Leu‐Phe dipeptide holds considerable potential in the pharmaceutical domain, indeed, Marchesan and coworkers found out that Leu‐Phe dipeptide could form a stable hydrogel in phosphate buffer [13]. Besides Leu‐Phe and Ile‐Phe dipeptides, no other dipeptide hydrogelator composed of natural amino acids has been reported to form a stable hydrogel. These hydrogels are characterized by high mechanical strength, self‐healing properties, and stability against proteases, making them suitable for biomedical applications. These hydrogels have been demonstrated to support three‐dimensional cell cultures, including HEK293T, HeLa, and HepG2 cells, indicating their potential in tissue engineering and regenerative medicine [14].

## 2 Materials and Methods

### 2.1 Chemicals and Reagents

HPLC‐grade methanol (MeOH) was purchased from Altium s.r.o. (Prague, Czech Republic). Analyte (Figure 1A) used in the study was obtained from LeapChem (Hong‐Kong, China).

### 2.2 Instrumentation

HPLC analyses were performed either on an LC‐20ADXR chromatograph (Shimadzu, Kyoto, Japan) or an Agilent 1100 (Agilent Technologies, Waldbronn, Germany) equipped with a precision pump, a degasser, an autosampler, a thermostatted column compartment, UV detector, and a data workstation running the Laboratory solution (LC‐20ADXR) or ChemStation (Agilent Technologies) software. Commercially available Chiralpak ZWIX(−)(CSP 1) and Chiralpak ZWIX(+)(CSP 2), and experimental ZWIX(−A)(CSP 3) and ZWIX(+A)(CSP 4) columns (150 × 3.0 mm I.D.; 3 µm; 120 Å pore size; from Chiral Technologies Europe, Illkirch, France (Figure 1B) were used. The mobile phase composed of MeOH/**H_2_O** (80/20, v/v). The flow rate was set at 0.3 mL min^−1^ and the column temperature at 20°C. The detection wavelength was 254 nm. Analytes were dissolved in methanol at the concentration of 1–2 mg mL^−1^. Chromatographic data was post‐processed with MS Excel (Microsoft, Redmond, WA, USA). Chromatograms and graphs were prepared in OriginPro 2019b (OriginLab Corporation, Northampton, MA, USA) and chemical structures were drawn in ChemDraw Professional (PerkinElmer, Waltham, MA, USA).

### 2.3 Molecular Modeling

The Maestro 13.5 graphical interface of the Schrödinger Suite 2024‐1 (Schrödinger, LLC, New York, NY, 2024) was used. As already used in previous work [8], a box was built with a 41.7 Å × 41.7 Å × 29 Å (*xyz* side length). In the present work, the procedure indicated an optimal box size of 41.7 Å × 41.7 Å × 26 Å (*xyz* side length). For a realistic reproduction of the stationary phase environment, four 3‐mercaptopropyl‐functionalized silanols (∼ 1.97 mol m^−2^), eight free silanols (∼ 8.0 mol m^−2^) and 45 silicon atoms were considered for each grafted selector (SO) unit (∼ 0.5 mol m^−2^), at the base of the box. All the silicon atoms and their bonded hydrogen atoms in the base layer were set frozen during the MD. The box was solvated with a mixture of MeOH/H_2_O (80/20, v/v) using the Packmol tool [15]. All the simulations with the two CSP systems were performed in the canonical ensemble at 298 K. The temperature in the simulation cell was maintained constant through use of a Nosé–Hoover thermostat [16, 17]. All the other parameters in the simulation study were left to default values in the Desmond Molecular Dynamics System (version 7.3, Schrödinger, LLC, New York, NY, 2023) present in the Schrödinger Suite 2024‐1 [18]. The optimized protocol consisted of a production run generating 4000 frames during the 1000 ns dynamics, with an integration time of 2 fs. All the frames of the trajectories were used to calculate the number and the type of interactions engaged, together with three energy values. In particular, three descriptors were analyzed: the relative conformational energies of the SO and the SA (namely SELF‐SO and SELF‐SA, obtained by subtracting the respective energy minima recorded along the trajectory from the conformational energy measured in the frame, in kcal mol^−1^), and the interaction energy between SO and SA (INTER, in kcal mol^−1^). A k‐mean clustering protocol using KNIME 4.0 software (KNIME GmbH, Konstanz, Germany) was used and applied to the matrix (one frame per row described by the energy values in three columns) to obtain five clusters to be plotted as bubble graphs.

## 3 Results and Discussion

### 3.1 Chromatographic Results

As previously stated, two dipeptides (Figure 1A) were investigated with the four *Cinchona*‐alkaloid based CSPs Chiralpak ZWIX(−)(CSP 1), Chiralpak ZWIX(+)(CSP 2), ZWIX(−A) (CSP 3) and ZWIX(+A) (CSP 4) (Figure 1B). They are versatile for direct enantiomer resolution of amino acids and many other ampholytic compounds by HPLC [19, 20, 21, 22, 23, 24, 25, 26, 27, 28]. The synergistic double ion‐pairing between the zwitterionic chiral selector and the analyte has been already identified in our previous studies [19, 29] as a driving force in the chiral recognition mechanisms. CSP 1 and 2 behave pseudo‐enantiomerically and provide the feature of reversing the EO of enantiomers by column switching. In an appropriate mobile phase medium, the chiral selector in CSP 1 or 2 is in a solvated zwitterionic state bearing a positively charged site at the nitrogen atom of the bicyclic quinuclidine and a negatively charged site at the level of sulfonic acid in the ACHSA ring. By the same principle, the ampholytic analyte undergoes a similar ionization process in the same mobile phase system. In these circumstances, strong coulombic attraction forces come into play for the synergic double ion‐pairing between the chiral selector and the analyte. It is expected that the analyte cation interacts with the anionic site of the chiral selector and the analyte anion interacts with the cationic site of the chiral selector via long range electrostatic forces. The enantiomer binding better inside the pocket of the chiral selector will form the more stable diastereomeric associate and will be more retained with respect to its antipode. However, the interaction of two zwitterionic species gives also rise to mutual repulsion. Therefore, the ZWIX columns can be used also under buffer free reversed phase conditions in accordance with the electrostatic attraction–repulsion model [20]. Undoubtedly, the mobile phase plays an essential role in such a double ion‐paring mechanism. It is supposed to be able to induce the expected ionizations of the chiral selector and the ampholytic analyte molecules, to have a suitable solvation power to all the ionized entities and at the same time offer convenient elution strength. Under such a context, the involvement of protic solvents in the mobile phase is recommended.

Consistent with the well‐known “pseudo‐enantiomeric” character of the two SOs due to the opposite configurations at C8 and C9, the studied analytes experienced the reversal of the EO, passing from one CSP to another [6, 30] (Table 1). Two typical chromatograms of the Leu‐Phe enantiomers obtained on CSP 3 and 4 are reported in the Supporting Information (Figures S1 and S2).

**TABLE 1:** Retention and enantioselectivity characteristics obtained for the analytes. The experimental conditions are detailed in section 2.2. Parameters *k*_1_ and *k*_2_ are the respective retention factors of the first and the second eluted enantiomer of each pair; α is the enantioselectivity (separation factor) of each pair of enantiomers; and EO represents the elution order (EO) of dipeptides.

Analyte	CSP	Chromatographic parameters			
		*k_1_*	*k_2_*	*α*	EO
Leu‐Phe‐OH	**1**	0.19	0.43	2.24	**(LL) < (DD)**
	**2**	0.69	1.43	2.07	**(DD) < (LL)**
	**3** ^a^	0.63	2.94	4.66	**(LL) < (DD)**
	**4** ^a^	0.77	2.39	3.11	**(DD) < (LL)**

^a^ The data on CSP 3 and 4 were obtained previously [32].

### 3.2 Molecular Modeling Methodological Approach

The methodological approach employed in this study was recently developed [10] by some authors of the present work. In line with the previous study, this investigation also assessed the impact of two experimental variables on the adherence of computational and chromatographic data: the duration of the MD production run and the frequency of recorded trajectory snapshots. Once the optimal settings were established, the trajectory was utilized to calculate some specific molecular descriptors that may elucidate the nature and significance of the different interactions occurring between the SAs and CSPs.

The results of the previous study unequivocally demonstrated that the enantiorecognition mechanism can be clarified at a molecular level through the investigation of the interaction energies of the SO–SA associates (referred to as INTER), which thereby exclude the contributions by the other achiral elements of the CSP that could obscure informative data.

Moreover, for each trajectory snapshot, the conformational energy of the sole SO and SA (referred to as SELF‐SO and SELF‐SA, respectively) in each frame was calculated, both in the absence or presence of SO–SA interactions. Consequently, every MD trajectory snapshot was described by these three energy values, which were used to create a matrix. This matrix was then submitted to the *k*‐means clustering procedure to identify families of SO–SA complexes sharing similar energy profiles, thus allowing the rationalization of the EO of enantiomers.

#### 3.2.1 Method Development and Optimization

MD simulations provide a dynamic representation of the interactions occurring between the SA enantiomers and the SO. Accordingly, a multitude of energy‐related data are collected along the MD trajectories.

Previous studies [8, 10, 29] have readily suggested the importance of an a priori fine‐tuning of the computational settings to gain information about the network of interactions at the basis of the enantiorecognition mechanism as well as the different magnitude of stereoselective interactions. By establishing a reliable analysis workflow for MD trajectories, we were able to extrapolate the necessary data to understand the driving forces engaged in the enantiorecognition mechanism with different analytes and different kinds of CSPs ultimately obtaining deeper insights into the interpretation of the experimental EO.

In this paper, we initially set a 41.7 Å × 41.7 Å × 29 Å (*xyz* side length) simulation box, as previously described in [8, 10, 24], filled with the virtual MeOH/H_2_O (80/20, v/v) mobile phase. The solvation of the simulation box was conducted in accordance with the established protocols, thus ensuring the reliable reproduction of the overall chromatographic system utilized in the HPLC runs. In addition, a duration of 500 ns collecting a total of 2000 frames was set.

By the aid of the Maestro interface present in the Schrödinger Suite 2024‐1 [17], eight systems were built, one per each enantiomer with the four CSPs. Each virtual stationary phase included the SA, the silicon atoms, the free silanols, those functionalized with the 3‐mercaptopropylgroups as well as those grafted with the SO units. The virtual chromatographic setting was then submitted to an MD protocol, which was then optimized in terms of simulation time, trajectory frames to collect, and box dimensions.

Following the conclusion of the dynamic run, the analyses of the three energies, as well as the type and number of interactions (π–cation‐, H‐bond, salt bridge, π–π stacking), were elaborated through a KNIME [31] workflow, providing a data matrix as output. It is important to highlight that SELF‐SA and SELF‐SO assume positive values, considering zero their respective minimum energy status. This procedure resulted in the generation of a new data matrix, where each row represented a fully described trajectory frame, with indications about the SO–SA binding mode (number and type of established interactions registered) in the snapshot, together with the key energy aspects describing the same SO–SA associate. Elaborating the data through the KNIME workflow, it readily emerged that most of the frames were not informative owing to the absence of interactions between SA and SO. In order to address this issue, we investigated the possibility of improving this result by changing some simulation settings in terms of MD duration, sampling frequency of the frames, and box size. Along this line, a longer run of 1000 ns was initially simulated by collecting a higher number of frames, this time 4000 frames. As expected, the percentage of informative frames was found to improve (data not shown). In an attempt to further enhance these results, the box size was optimized, with the *z* dimension of the box being reduced from 29 to 26 Å. This modification was applied exclusively to the space designated for the placement of solvent molecules.

The final optimized settings comprised a box with dimensions of 41.7 Å × 41.7 Å × 26 Å (*xyz* side length), with a production run of 1000 ns enabling the capture of 4000 frames. The simulation box should be of sufficient size to accurately reproduce the chromatographic environment, allowing the “catch and release” of the SA by the SO. The use of oversized boxes could have a negatively impactful effect on the simulation, as this could result in a limited number of frames regarding the SO–SA associates. Conversely, a box that is too small could unrealistically promote SO–SA association events. Therefore, optimizing the box dimensions is a quintessential step to enhance the adherence with the real system.

The KNIME workflow facilitated the calculation and statistical analysis of all the aforementioned descriptors. Furthermore, to ascertain whether a SA can form SO–SA associates characterized by analogous conformational energy and INTER values, a *k*‐means clustering protocol was implemented within the KNIME workflow. This procedure generates five clusters of different populations for each trajectory, which can be represented as bubble graphs. This type of representation has already proved instrumental in identifying the overall distribution (i.e. population) of the SO–SA associates of each analyte and their corresponding energy profile [7, 8, 10, 29].

#### 3.2.2 Investigation of the Enantiomeric Elution Order by Interaction Monitoring

In order to determine whether the more strongly retained enantiomer experiences more SO–SA interactions than the less strongly retained enantiomer, an initial examination of the MD results was carried out. This type of analysis helps us to understand and discriminate among different types of interactions, and to identify those most relevant ones in ruling enantiomer retention. Therefore, it is of primary importance to consider the total number of interactions recorded (Table 2) for each type of interaction as it represents a key point to understand the relative weight of that specific interaction in the binding modes explored by the single enantiomer.

**TABLE 2:** Number and type of interactions engaged by the analytes (SAs) with the chiral selectors (SOs) during the molecular dynamic trajectory.

CSP	Analyte	SO–SA Interactions ‐ Tot (*N*)				
(EO)		Aromatic H‐Bond	π–cation	H‐bond	Salt bridge	π–π
**CSP 1**	DD‐Leu‐Phe‐OH	222	116	1481	910	28
**(LL < DD)**	LL‐Leu‐Phe‐OH	69	77	265	220	61
**CSP 2**	DD‐Leu‐Phe‐OH	43	37	238	165	29
**(DD < LL)**	LL‐Leu‐Phe‐OH	87	57	318	237	37
**CSP 3**	DD‐Leu‐Phe‐OH	330	281	1410	913	44
**(LL < DD)**	LL‐Leu‐Phe‐OH	111	204	479	360	160
**CSP 4**	DD‐Leu‐Phe‐OH	88	86	331	280	39
**(DD < LL)**	LL‐Leu‐Phe‐OH	126	204	536	498	67

Looking at the data listed in Table 2, it is clear that: (i) for the QD‐type CSP 1 and 3, only the number of π–π stacking interactions engaged by the two enantiomers is not in agreement with the experimental EO of the enantiomers; (ii) instead, a full correlation occurs with the QN‐type CSP 2 and 4; (iii) for CSP 1 and 3, the more abundant SA–SO interactions are the H‐bonds and the salt bridges; (iv) these interactions are extraordinarily enantioselective (large differences between the calculated contacts); (v) with respect to the QD‐type CSPs, a comparatively smaller difference in the number of enantiomeric interactions with the SO units is calculated with the QN‐type CSP 2 and 4.

The higher chromatographic enantioselectivities obtained with CSP 1 and 3 over CSP 2 and 4 are fully consistent with the pronounced differences in terms of H‐bond and salt bridge interactions. In addition, the data in Table 2 clearly show that the stereoselectivity of CSP 1 and 3 is largely due to these interactions, making the effect of π–π stacking interactions in the enantiorecognition mechanism negligible. The differential contribution of these interactions to the overall stereoselective event is due to the different conformation of the binding cleft imposed by the absolute configuration at C8 and C9. Based on the above, it can be speculated that a higher accessibility to the H‐bond and salt‐bridge interaction sites on the SO contributes to increase the enantioselectivity of the system and that these interactions always drive the enantiorecognition mechanism. The importance of the salt bridge interactions in the SO–SA binding is corroborated by the fact that the retention of the Leu‐Phe enantiomers was drastically decreased when ammonium acetate was added to the mobile phase at the concentration 50 mM [32]. Foreign ions shielded the fixed charges of the SO as well as those of the SA that resulted in diminished Coulombic interaction between them.

The above information was further enriched by a deeper analysis of the SO–SA binding modes explored during the MD trajectories. This analysis highlighted that the main difference among the CSPs reside in the number of H‐bonds engaged by the SAs with the sulfonic group of the employed SOs. In particular, the total number of H‐bonds with the anionic site of the SOs was 478, 379, 465, and 353 for CSP 1, 2, 3, and 4 respectively. It is also interesting to note, from the point of view of salt bridge interactions, that the dipeptide enantiomers almost always interacted with the SO units with a single charged functional group at a time, highlighting a single ion rather than a zwitterion interaction mechanism.

#### 3.2.3 The Role of the Energies in the Enantiodiscrimination Process

As already mentioned, all in silico evaluations were carried out considering 1:1 SA–SO associates, in which the ionic interaction takes place between the negatively charged SO sulfonic acid in the ACHSA ring and the N‐terminal of the SA, and the charged nitrogen in the bicyclic quinuclidine of the SO with the carboxy‐terminal of the SA. Following an MD protocol already applied in previous works [8, 10, 24], the enantioselective process was also investigated in terms of energy profiles. This was made possible by taking into account the INTER, SELF‐SO, and SELF‐SA descriptors mentioned above. More specifically, the plotting of INTER vs. SELF‐SO and INTER vs. SELF‐SA clusters allowed the construction of “bubble graphs” that helped to explain the enantioselective event. In the bubble graph representation, the more retained enantiomer should be the one characterized by more and/or highly populated bubble clusters located in the lower left corner of the plot. This distribution indicates an energetically more favorable SO–SA complex. In other work, the more retained enantiomer is expected to have more highly negative INTER and SELF‐SO and SELF‐SA values than the less retained one.

**FIGURE 2:**
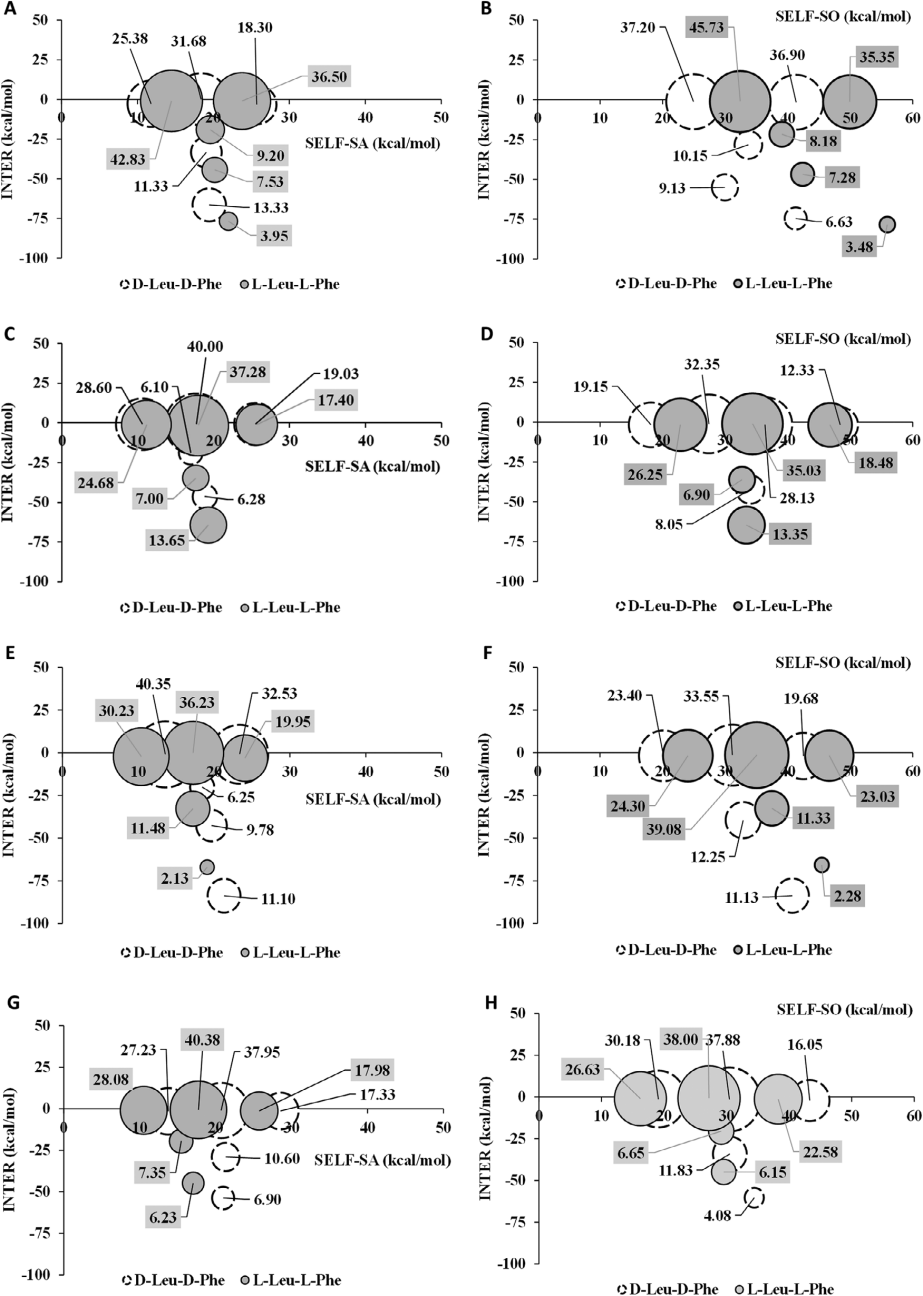
Bubble graphics of the class distribution resulting from the *k*‐means clustering protocol using the SELF‐SO, the SELF‐SA, and the INTER descriptors for dipeptides D‐leucine‐D‐phenylalanine and L‐leucine‐L‐phenylalanine. (A, B) are with CSP 1, (C, D) are with CSP 2, (E, F) are with CSP 3, and (G, H) are with CSP 4. The percentage of the relative population is reported near each bubble.

In strict accordance with the above considerations, the enantioselective mechanism was well explained by the bubble graphs shown in Figure 2. Correspondingly, the EO of the enantiomers (Tables 1 and 2) is consistently explained by the energy profiles and populations of the bubble clusters, with the logical exclusion of the bubbles with the cluster centroid characterized by an INTER value lower than 10 kcal/mol. In general, the INTER values explain well the observed EO of the enantiomers. In addition, the population of more stable associates helps to support the experimental results. Interestingly, for the CSP 1 and 3, a contribution of the SO conformational energies (SELF‐SO) to the EO of enantiomers is readily apparent (Figure 2). Accordingly, the cluster disposition for the DD enantiomer populates the left part of the plot more than the clusters of the LL enantiomer.

### 3.3 Comparison of the Four Chiral Selectors

The differences between the four CSPs were also investigated using the two enantiomers as probes to monitor their interactions with the four SOs (Figure 1A) [33, 34, 35, 36, 37, 38, 39, 40].

**FIGURE 3:**
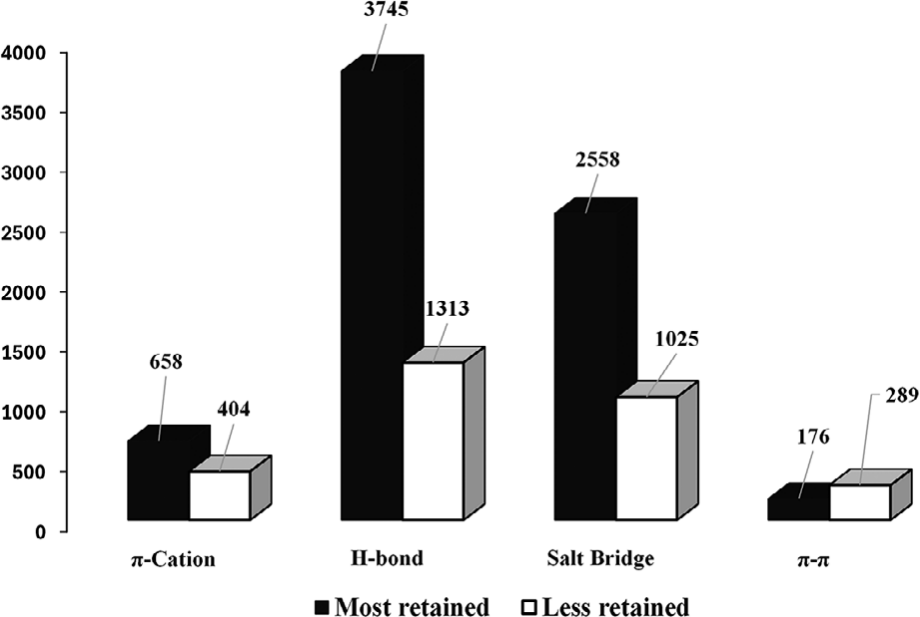
Histogram of the sum of the different types of interactions monitored on all analytes with the two CSPs.

A comparison of the data on the sum of all interactions recorded between the most and least retained enantiomer with all CSPs (Figure 3) showed that the most frequent SO–SA interactions were H‐bond and salt bridge interactions.

Since the SOs in the four CSPs have a diastereomeric relationship, it is conceivable that their different conformation may influence the frequency of each type of interaction in the context of the SO–SA association process.

**FIGURE 4:**
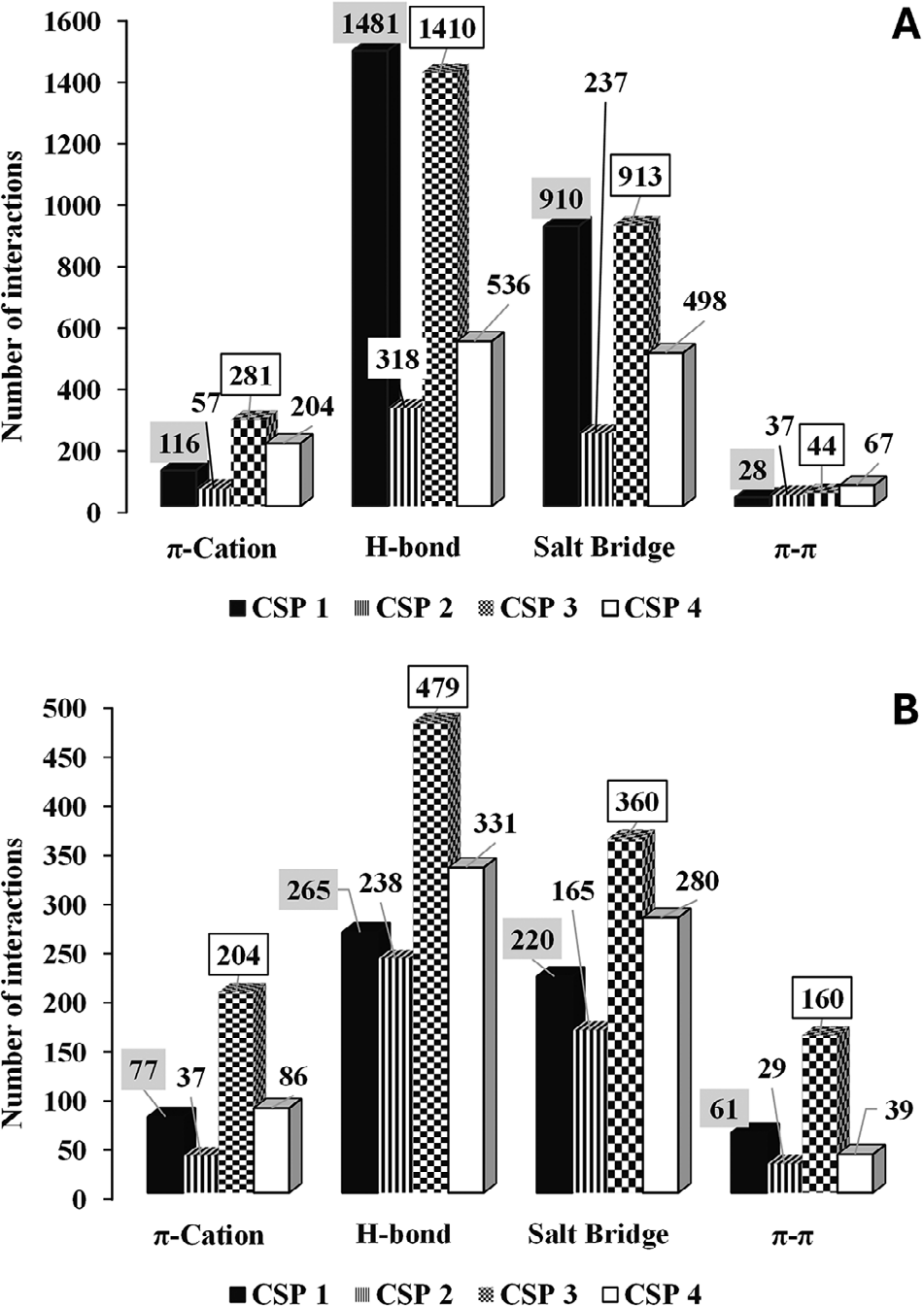
Histogram of the sum of the different types of interactions monitored for the most (top) and the less (bottom) retained enantiomers with CSP 1 and 2.

To gain further insight and identify differences between the SOs, all different types of interactions for the most and least retained enantiomers were calculated for all CSPs and presented as bar plots in Figure 4. As expected, and shown here in detail, the most retained analytes exhibit a greater number of interactions than the less retained enantiomers, particularly in terms of H‐bond and salt bridge interactions, both of which play a prominent role in the enantiorecognition mechanism. This was particularly evident for CSP 1 and 3, which showed higher enantioselectivities than their pseudo‐enantiomeric variants CSP 2 and 4 (Table 1).

## 4 Conclusions

In this study, we have successfully optimized an in silico MD method to investigate the enantiorecognition mechanism taking place in four different zwitterionic‐based chiral columns with respect to the DD/LL‐Leu‐Phe dipeptide. The simulation box size, run time, and number of frames were optimized to generate MD trajectories as informative as possible. In addition, the type and number of interactions of the SAs with the SOs, the SO and SA conformational energies and the SO–SA interaction energies were monitored.

These analyses were in complete agreement with the experimental EO, providing insights into the driving forces involved in the enantiorecognition mechanism. In particular, the primary interactions facilitating the SO–SA association were confirmed to be salt bridges and hydrogen bonds. In addition, π–π and π–cation interactions were identified as complementary interactions that further enhance the stability of the SO–SA complexes. In terms of the energy profile, as expected, the SO–SA interaction energy was identified as a key factor in all cases.

## Author Contributions

Ina Varfaj: Conceptualization, methodology, investigation, writing—original draft, validation, visualization, and writing—review & editing. Roccaldo Sardella: Conceptualization, methodology, Resources, Writing—review & editing. Yana A. Klimova: Conceptualization, methodology, investigation. Resources and writing—review & editing. Leonid D. Asnin: Conceptualization, methodology, investigation, Writing—review & editing. Michael Kohout: Conceptualization, methodology, investigation, writing—original draft, validation, visualization, and writing—review & editing, Resources. Andrea Carotti: Michael Kohout: Conceptualization, methodology, investigation, writing—original draft, validation, visualization, and writing—review & editing, Resources.

## Conflicts of Interest

The author declares no conflicts of interest.

## Supporting information




**Supporting File 1**: jssc70220‐sup‐0001‐SuppMat.docx.

## Data Availability

The data that support the findings of this study are available from the corresponding author upon reasonable request.
