# Supplementary material for: Application of Molecular Dynamic Simulation in the Enantiorecognition Mechanism of the Pharmaceutically Relevant Leu‐Phe Dipeptides With Four Zwitterionic Chiral Stationary Phases
Source: J Sep Sci. 2025 Jul 8;48(7):e70220. doi: 10.1002/jssc.70220 (PMC12238692; doi:10.1002/jssc.70220)
Supplement: Supplementary file 1 — Supporting File 1: jssc70220‐sup‐0001‐SuppMat.docx. [file JSSC-48-e70220-s001.docx]

**Supplementary Material**

**Application of molecular dynamic simulation in the enantiorecognition mechanism of the pharmaceutically relevant Leu-Phe dipeptides with four zwitterionic Chiral Stationary Phases**

Ina Varfaj^1^, Roccaldo Sardella^1^, Yana A. Klimova^2^, Leonid D. Asnin^2^, Michael Kohout^3^*, Andrea Carotti^1^*

*^1^ Department of Pharmaceutical Sciences, University of Perugia, Via Fabretti 48, 06123 Perugia, Italy*

*^2^ Perm National Research Polytechnic University, 29 Komsomolsky Al., Perm 614990, Russia*

*^3^ Department of Organic Chemistry, University of Chemistry and Technology, Prague 6, Czech Republic*

Corresponding authors: Prof. Andrea Carotti, Department of Pharmaceutical Sciences, University of Perugia, Via Fabretti 48, 06123 Perugia, Italy; email: [andrea.carotti@unipg.it](mailto:andrea.carotti@unipg.it); Prof. Michal Kohout, Department of Organic Chemistry, University of Chemistry and Technology, Prague 6, Czech Republic, email: [Michal.Kohout@vscht.cz](mailto:Michal.Kohout@vscht.cz)

Table of Contents

- Chromatograms obtained for CSP 3 and CSP 4 (Fig. S1-S2) S2

**Figure S1.** Chromatogram of the Leu-Phe enantiomers on experimental ZWIX(−A)™(CSP 3) column (150 × 3.0 mm I.D.; 3 µm; 120 Å pore size. The mobile phase composed of MeOH/H_2_O (80/20, v/v). The flow rate was set at 0.3 ml min^−1^ and the column temperature at 20 °C. The detection wavelength was 254 nm. Analytes were dissolved in methanol at the concentration of 1-2 mg ml^−1^.





**Figure S2.** Chromatogram of the Leu-Phe enantiomers on experimental ZWIX(+A)™(CSP 4) column (150 × 3.0 mm I.D.; 3 µm; 120 Å pore size. The mobile phase composed of MeOH/H_2_O (80/20, v/v). The flow rate was set at 0.3 ml min^−1^ and the column temperature at 20 °C. The detection wavelength was 254 nm. Analytes were dissolved in methanol at the concentration of 1-2 mg ml^−1^.

**

**
